# Hyaluronic Acid and Skin: Its Role in Aging and Wound-Healing Processes

**DOI:** 10.3390/gels11040281

**Published:** 2025-04-09

**Authors:** Natalia Chylińska, Mateusz Maciejczyk

**Affiliations:** 1 Independent Laboratory of Cosmetology, Medical University of Białystok, Akademicka 3, 15-267 Bialystok, Poland; 2 Department of Hygiene, Epidemiology and Ergonomics, Medical University of Białystok, Mickiewicza 2c, 15-022 Bialystok, Poland; mat.maciejczyk@gmail.com

**Keywords:** hyaluronic acid, skin aging, wound healing

## Abstract

Hyaluronic acid (HA) is a linear, unbranched polysaccharide classified as a glycosaminoglycan. While HA is found in various tissues throughout the body, over half of its total proportion is found in the skin. The role of HA in the skin is complex and multifaceted. HA maintains proper hydration, elasticity, and skin firmness, serving as a key extracellular matrix (ECM) component. With age, HA production gradually decreases, leading to reduced water-binding capacity, drier and less elastic skin, and the formation of wrinkles. Additionally, HA plays an active role in the wound-healing process at every stage. This review summarizes the current background knowledge about the role of HA in skin aging and wound healing. We discuss the latest applications of HA in aging prevention, including anti-aging formulations, nutricosmetics, microneedles, nanoparticles, HA-based fillers, and skin biostimulators. Furthermore, we explore various HA-based dressings used in wound treatment, such as hydrogels, sponges, membranes, and films.

## 1. Introduction

Hyaluronic acid (HA) has diverse and unique properties [1,2]. It occurs naturally in all living organisms, commonly as a sodium salt (sodium hyaluronate) [3]. It was first discovered and isolated from the vitreous body of a bovine eye in 1934 by American scientists Karl Meyer and John Palmer [4]. The name HA originates from a combination of the words hyaloid (vitreous body) and uronic acid [4,5]. HA is a significant component of the human body’s extracellular matrix (ECM). Over 50% of the total HA content in the human body is found in the skin [4,6,7]. HA is also found in the synovial membrane, articular cartilage, the vitreous body of the eye, blood vessel walls, skeletal muscle tissue, the umbilical cord, and internal organs, primarily in the kidneys and lungs [4,7]. HA performs several important biological functions [8]. Above all, it ensures the proper functioning of joints and tendons, reduces inflammation, regulates angiogenesis and tissue neovascularization, and plays a role in morphogenesis, wound healing, and cellular signaling [5,8,9,10,11,12]. In human skin, HA is a key substance that fills the ECM and is responsible for maintaining proper hydration, elasticity, and firmness of the skin [7,13,14,15].

The level of HA in the human body is not constant [7,15]. With the advancement of aging and the influence of environmental factors, especially excessive exposure to sunlight, the amount of HA significantly decreases, resulting in a loss of skin hydration, changes in its topography, the formation of wrinkles, sagging, limited joint mobility, and impaired healing of burns, scars, and wounds [7,15,16,17]. Therefore, external delivery is increasingly being used to supplement HA’s deficiencies [15]. Depending on the purpose and location of the therapy, HA may be administered in the form of intra-articular injections, as a wrinkle and skin filler, through oral supplementation, or for external application, most commonly in the form of creams, serums, or eye drops [1,14,18]. HA is also commonly used as a delivery system for drugs and active substances as well as a substance that increases their penetration through the stratum corneum [19]. HA-based preparations have been widely applied in medicine, both in aesthetic and therapeutic capacities [4,18]. Due to its versatile effects, HA is widely used in orthopedics, ophthalmology, otolaryngology, gynecology, rheumatology, aesthetic medicine, and cosmetology [4,18]. HA’s wide range of properties and numerous applications continue to drive research into understanding the exact molecular mechanism of HA’s action [20,21].

In the 1950s, HA was extracted from human umbilical cords and animal tissues, mainly from rooster combs, synovial fluid, and the skin of marine animals. Despite production optimization, these sources of HA posed significant contamination risks and often triggered allergic reactions [4]. Therefore, ever since, efforts have been made to find alternative methods for its extraction. Following the development of biotechnological methods, HA is now also produced through the fermentation of Gram-positive streptococci, namely, *Streptococcus equi*, and Gram-negative bacteria, i.e., *Pasteurella multocida* [22,23,24,25].

HA is a key material used for advanced biomedical and cosmetic applications. This is due to HA’s several properties, such as biocompatibility, biodegradability, and the ability to be chemically modified. At the same time, the unique characteristics of HA make it a promising substrate for developing new therapeutic strategies. Every year, more and more data are emerging on new potential applications in tissue engineering and regenerative medicine, transdermal drug delivery systems, cosmetic preparations, and tissue fillers. This review aims to summarize the current knowledge on the role of HA in the skin-aging process and wound healing and present the latest applications of HA in regenerative medicine, anti-aging therapies, aesthetics, and cosmetology.

## 2. Structure, Properties, and Biological Functions of Hyaluronic Acid

Hyaluronic acid (HA) is a linear, unbranched polysaccharide. Its chemical structure is the same in the human body and most bacteria. The hyaluronic acid molecule comprises repeating disaccharide units of D-glucuronic acid and N-acetyl-D-glucosamine, connected by β-1,4 and β-1,3 glycosidic bonds [4,26,27,28]. The excellent water solubility of HA polymers is attributed to their β-1,3 glycosidic bonds [29]. It has been shown that HA molecules adopt a double left-handed helical structure in aqueous solutions, with hydrogen bonds being responsible for its stabilization [4,30].

Hyaluronic acid (HA) belongs to the group of glycosaminoglycans [5,6]. Other group members include heparin and sulfates: heparan sulfate, dermatan sulfate, keratan sulfate, and chondroitin sulfate [4]. These are polyanionic compounds that contain acidic sulfate groups. Glycosaminoglycans are bonded to proteins, forming proteoglycans (e.g., beta glycan, aggrecan, syndecan, perlecan, and decorin) [31,32,33]. One of the most important properties of glycosaminoglycans is their strong hydrophilicity and ability to increase their volume in an aqueous environment [34,35,36,37,38,39]. HA’s high osmotic activity and anionic structure enable it to bind large amounts of water [4,40,41,42]. The polymeric nature of HA, on the other hand, ensures good viscoelastic properties [43]. In addition to the mentioned characteristics, HA differs significantly from other glycosaminoglycans. Primarily, HA is a non-sulfated biopolymer [7,10]. Among all glycosaminoglycans, HA is the only one that does not form covalent bonds with the protein core [7]. Despite this, proteoglycans may bind to HA, forming proteoglycan aggregates. By attaching to HA, these aggregates fill the spaces of the extracellular matrix as an amorphous gel [7]. Unlike most glycosaminoglycans, HA is not synthesized by the Golgi apparatus and has a minimal negative charge density, as it contains only one carboxyl group [4,34,44]. HA can be chemically modified via conjugation or crosslinking reactions. These processes result in HA derivatives with improved natural retention and reduced tissue clearance compared to native HA [45,46]. Many HA derivatives with greater biological stability have been developed. One of them is a derivative of ethylenediamine (EDA) and octadecylamine (C_18_-NH_2_), referred to as HA-EDA-C_18_ [46].

HA’s physicochemical and biological properties largely depend on its molecular weight and the size of the molecule (Table 1) [4,37]. The greater the molecular weight of HA, the better its viscoelastic properties [37]. The naturally occurring HA in the human body has a molecular weight in the range of 10^5^–10^7^ Da [4,8,47]. The molecular weight of HA varies depending on its location in tissues [48]. For example, the molecular weight of HA in synovial fluid is between 6000–7000 kDa, while in joint fluid it is between 3000 and 5000 kDa [37]. HA in the epidermis constitutes approximately 0.1 mg/g of wet tissue, while in the dermis, it makes up 0.5 mg/g [29,49]. High-molecular-weight HA (HMW-HA) has a molecular weight of ≥1000 kDa, medium-molecular-weight HA (MMW-HA) has a molecular weight ranging from 250 to 1000 kDa, and low-molecular-weight HA (LMW-HA) has a molecular weight ranging from 10 to 250 kDa [50]. It has been shown that HMW-HA can have anti-inflammatory and anti-angiogenic effects [49,50,51]. The anti-angiogenic activity of HMW-HA results from its ability to inhibit the migration and differentiation of endothelial cells [52]. HMW-HA also reduces the production of interleukin 1-β (IL1-β), inhibits the activation of the toll-like receptor 4 (TLR4) signaling pathway, and suppresses the activity of metalloproteinase 9 (MMP9), the elevated expression of which is observed in inflammatory responses [53]. Additionally, HMW-HA increases the expression of transforming growth factor-beta 3 (TGF-β3), which inhibits the scar formation process [54,55]. MMW-HA, on the other hand, enhances the expression of inflammatory factors such as tumor necrosis factor (TNF-α) and interleukins 6 (IL-6) and 1β (IL-1β) [49]. LMW-HA also stimulates the production of inflammatory cytokines and cell proliferation and exhibits proangiogenic activity through interaction with the endothelial lymphatic vessel receptor 1. It promotes lymphatic endothelial cells (LECs), which are crucial in lymphangiogenesis [48,56,57].

The biological properties of HA also arise from its interactions with binding proteins known as hyaladherins [4,61]. The proteins that bind HA include the transmembrane glycoprotein CD44, the receptor RHAMM (receptor for hyaluronan-mediated motility), lymphatic vessel endothelial hyaluronan receptor-1, the intracellular adhesion molecule ICAM-1, and TLRs (toll-like receptors) [7,61,62,63,64]. The main receptor responsible for the internalization and degradation of HA is CD44 [4,65]. CD44 is found in most cells but not red blood cells [7]. CD44 binds to HA on the cell surface [65]. The CD44-HA interaction plays a crucial role in many cellular processes, including cell migration, adhesion, and the activation and extravasation of lymphocytes to areas affected by inflammation [7,66]. It has been shown that the binding of CD44 to HA activates Rho GTPase proteins. Rho GTPases play a key role in remodeling the actin cytoskeleton, which is necessary for the migration and adhesion of keratinocytes. Additionally, the activation of Rho GTPase promotes the mobilization of intracellular calcium, which is essential for keratinocyte differentiation [67]. RHAMM regulates cell-signaling pathways and stimulates the migration and proliferation of both non-cancerous and cancerous cells [62,67,68]. It has been shown that the RHAMM-HA interaction plays a role in the mobility of cells stimulated by transforming growth factor beta (TGF-β) [67]. Lymphatic vessel endothelial hyaluronan receptor-1 is involved in the degradation of HA and its uptake by lymphatic endothelial cells, followed by its transport to lymph [4,62,69]. ICAM-1, also known as the CD54 receptor, mediates the activation of inflammatory processes and is involved in releasing HA from bodily fluids and plasma [26,62]. HA also regulates the activity of TLRs that recognize bacterial components such as lipopolysaccharides and lipopeptides and subsequently initiate the innate immune response [4,70].

## 3. The Role of Hyaluronic Acid (HA) in Skin Aging

### 3.1. Skin Aging

The skin is the human body’s largest organ, performing many physiological functions [39,71,72,73]. It is a physical barrier protecting internal organs from the harmful effects of environmental factors, such as mechanical, physical, and chemical damage [39,71,72,73]. An important component of the skin is the extracellular matrix (ECM), which fills the space between cells and is responsible for maintaining tissue integrity [74,75]. The composition of the ECM is highly varied and depends on the type of tissue and its location [76,77]. The ECM contains insoluble fibrous proteins, such as collagen and elastin, as well as glycoproteins, fibronectin, and laminin [74,75,76]. Collagen is one of the most important components of the skin’s framework. In the dermis, it forms a network of loose collagen fibers that provide structural integrity, elasticity, and strength [75]. Collagen also plays a crucial role in wound healing, facilitating the migration and proliferation of new cells, regulating inflammation, and stimulating the regeneration of damaged tissues [75,78,79,80]. Elastic fiber, on the other hand, is essential for tissue repair and allows the skin to maintain its elasticity, resilience, and resistance to mechanical injuries [77,78]. Fibronectin accelerates wound healing by facilitating cell adhesion and migration [75,81]. Laminin, on the other hand, is a key component of the cell membrane, playing an important role in maintaining skin integrity [82]. The skin matrix also contains transmembrane receptors called integrins that are involved in signal transduction and form intercellular connections and connections between cells and the ECM. Integrins bind to the cytoskeletons of skin cells, thereby serving a structural function [76,83,84,85,86]. The main component of the ECM is HA, which is characterized by its strong hydrophilic properties [7]. HA is responsible for maintaining the structural integrity of the ECM and tissue hydration and plays an important role in the wound-healing process [7,87]. HA gives the skin cushioning properties by forming network-like connections with ECM proteins [88]. As the amount of HA decreases, the skin’s susceptibility to mechanical damage increases [89]. HA, collagen, and elastin production occur in fibroblasts and keratinocytes, the most important cell types in the dermis [90]. The presence of appropriate amounts of HA, collagen, and elastin in the skin ensures its proper function and a youthful appearance [90].

The skin also plays immunological roles. It forms an extensive network of cells such as keratinocytes, fibroblasts, endothelial cells, leukocytes, macrophages, mast cells, and Langerhans cells, as well as many soluble antimicrobial peptides, cytokines, and chemokines, that together create a unique immune system of the skin [91]. As a result, the skin regulates many important processes, such as angiogenesis, inflammation, and wound healing while also preventing pathogenic microorganism entry [92].

As we age, the efficiency of our cellular repair systems decreases, and the function of the immune system of the skin becomes less effective [93,94,95]. As a result of aging, there is a decrease in the number of skin fibroblasts and their function [90]. They synthesize smaller amounts of collagen, elastin, and HA, resulting in wrinkles, decreased elasticity and firmness of the skin, and excessive dryness [7,15,90]. The pathomechanism of skin aging is explained by various theories, often overlapping [95,96]. Nevertheless, skin aging may be intrinsic or extrinsic [96,97]. Intrinsic skin aging, often called chronological or endogenous aging, is inevitable and driven by physiological changes that occur in the skin as we age [88,89]. It is primarily regulated by genetic and hormonal factors [96]. The second type of skin aging is extrinsic aging or exogenous aging. This type of aging is influenced by environmental factors such as ultraviolet (UV) radiation, air pollution, tobacco smoke, extreme temperatures, and humidity [96]. The sun’s ultraviolet (UV) radiation causes premature skin aging, referred to as photoaging. This results in the degeneration and photo-damage of the epidermis and dermis, leading to skin pigmentation disorders, excessive keratinization of the epidermis, deep wrinkles, skin laxity, and even the development of skin cancer [96,98,99].

An important factor accelerating premature skin aging is oxidative stress [99]. Oxidative stress is responsible for the development of many age-related diseases, such as neurodegenerative diseases, cardiovascular diseases, degenerative joint changes, chronic inflammation, and diabetes [100,101,102,103]. Oxidative stress is defined as a disruption of the homeostasis between reactive oxygen species (ROS), such as hydrogen peroxide (H_2_O_2_), singlet oxygen (^1^O_2_), superoxide radicals (O_2_^•−^), hydroxyl radicals (OH), and the antioxidant properties of the body [103,104,105]. ROS are highly reactive molecules that can oxidize cellular components of the skin, leading to protein damage, lipid peroxidation, DNA damage and mutations, and the activation of inflammatory pathways, and they contribute to skin aging, the development of cancer, and the impairment of wound healing [106,107,108]. Factors that increase ROS production in the skin include UV radiation, air pollutants such as ozone and particulate matter, cigarette smoke, a poor diet, and excessive alcohol consumption [107,109,110]. The overproduction of ROS increases the activity of matrix metalloproteinases (MMPs), which are responsible for remodeling the skin’s ECM [13]. The primary sources of MMPs in the skin are fibroblasts and keratinocytes. However, MMPs may also be produced by immune and endothelial cells [111]. MMPs carry out many important functions in the skin, including participating in angiogenesis, wound healing, tissue repair, and ECM remodeling [112]. However, the overproduction of MMPs may damage ECM proteins such as collagen and elastin [113]. In UV-damaged skin, the expression of MMPs, particularly MMP1 and MMP12, significantly increases [13,112]. The primary protease that degrades type I and III collagen is collagenase 1, also known as MMP1 [13]. On the other hand, macrophage elastase MMP12 is capable of degrading elastin and other ECM components, including laminin, fibronectin, and type I and IV collagen [13]. The activity of MMPs is regulated by specific tissue inhibitors of metalloproteinases (TIMPs). TIMPs include proteases (TIMP1, TIMP2, TIMP3, and TIMP4), the primary function of which is to inhibit the activity of MMPs [114,115]. Excessive MMP activity relative to that of TIMPs causes disturbances in tissue remodeling [95,111]. Skin aging also influences protein glycation—a non-enzymatic reaction between sugars and proteins. As a result, advanced glycation end-products (AGEs) are formed, which are involved in oxidative stress and inflammation. AGEs interact with the ECM and damage the structural proteins of the skin [95,116]. AGEs bind to the receptor for advanced glycation end-products (RAGE), which plays a crucial role in tissue damage and the progression of various diseases. The interaction between AGEs and RAGE activates the nuclear transcription factor NF-kappa B (NF-κB) signaling pathway, leading to increased ROS production and exacerbating oxidative stress. This promotes inflammation and cellular damage [95,117].

### 3.2. Hyaluronic Acid in Skin Aging

HA, as the main component of the ECM, plays a key role in skin aging. It is estimated that a 70 kg human body contains as much as 15 g of HA in the form of sodium hyaluronate, with more than half of the HA reserves located in the skin [4]. Approximately one-third of the total HA pool undergoes degradation and is replaced by newly synthesized HA [118]. Skin HA is produced by enzymatic proteins called hyaluronan synthases (HASs) in two types of cells: fibroblasts and keratinocytes [13,15,39,118]. There are three subtypes of hyaluronan synthases (HAS1, HAS2, and HAS3), each involved in the synthesis of HA with different chain lengths [15,39,119,120]. It is worth noting that fibroblasts primarily use the HAS2 synthase, while epidermal keratinocytes use both HAS2 and HAS3 to an equal extent [15]. The HAS1 and HAS2 proteins are responsible for the production of HMW-HA, while HAS3 is responsible for the production of LMW-HA [121].

Interestingly, temperature significantly affects the degradation and synthesis kinetics of HA. Elevated temperatures accelerate HA degradation through increasing the activity of enzymes such as hyaluronidase [122,123]. At the same time, heating can reduce the activity of HA synthases by destabilizing their structures and decreasing HA production [122,124]. One study showed that after 24 h of exposure to high temperatures and pH levels, the molecular weight of HA decreased from 753 kDa to 36 kDa [122].

Until recently, it was believed that HA was not present in the epidermis [39]. Following the development of histochemical analysis techniques, HA’s presence in this skin layer was confirmed [39]. However, the amount of HA in the epidermis is significantly lower compared to that in the dermis [7,125]. The related literature data also indicate the different localization of hyaluronan in the individual layers of the epidermis [126,127]. In most of the related studies, it has been suggested that HA is not present in the stratum corneum of the epidermis [126]. Tammi et al. demonstrated that hyaluronan is primarily localized in the middle prickle layer. It is not found in the granular and horny layers, with only a small amount of HA existing in the basal layer [126]. Sakai et al. studied the expression of mRNA of HAS1 and HAS2 synthases in mouse skin using mRNA in situ hybridization. They found that both mRNAs are expressed in the granular and horny layers of the epidermis [128].

In the dermis, HA is found both in the papillary and reticular layers, with a greater amount found in the papillary layer [7,129]. Adiponectin is responsible for stimulating HA synthesis in dermal fibroblasts. Adiponectin is a cytokine produced by adipose tissue cells and sebaceous glands. It has been shown that skin fibroblasts possess specific adiponectin receptors. Adiponectin, through the AMP-activated protein kinase (AMPK) pathway and the peroxisome proliferator-activated receptor-α (PPARα), regulates HA production by potentiating the activity of HAS2 synthase [129,130,131].

HA is highly metabolically active; its synthesis is accompanied by a continuous process of its breakdown [125]. It has been found that the rate of HA degradation depends on HA’s location. The biological half-life of HA is 3–5 min in the blood, approximately 12 h in the skin, and 1–3 weeks in cartilage [7,125]. The degradation of HA in the skin occurs as a result of the action of hyaluronidase, a hydrolytic enzyme that depolymerizes HA, or through the action of ROS [4,125]. Six types of hyaluronidases have been identified in the human body: hyaluronidase-1, hyaluronidase-2, hyaluronidase-3, hyaluronidase-4, and sperm adhesion molecule 1 [4,132,133]. The greatest hydrolytic activity is primarily exhibited by hyaluronidase-1 and hyaluronidase-2 [132,133]. Hyaluronidase enzymes break down HA chains into smaller fragments that are then further cleaved by β-D-glucuronidase and β-N-acetyl-hexosaminidase [4].

In young skin, the amount of HA is optimal, which ensures good elasticity, firmness, and proper hydration levels. However, the physiological loss of HA begins quite early, with the age of 25 generally considered the threshold [134]. Longas et al. showed that the average HA concentration in the skin is 0.3 mg/g of wet tissue in the 19–47 age group. This concentration decreases with age, reaching 0.15 mg/g of wet tissue at the age of 60 and 0.07 mg/g of wet tissue in 75-year-olds [135]. Interestingly, HA is homogeneously distributed in young skin, forming a complex network connected to collagen and elastin fibers. In senile skin, HA deposits were absent in the intercellular and pericellular areas of the dermis, indicating a loss of structural integrity [136]. There is also a lack of data on the impact of skin aging on the molecular weight of HA. However, Holmes et al. revealed a gradual decrease in the size of HA polymers in articular cartilage with age. The molecular weight of HA was 2.0–3.0 × 10^6^ Da in the articular cartilage of 2.5-year-old children, while in a group of 86-year-olds, it was only 0.5 × 10^6^ Da. This indicates a progressive reduction in polymer size associated with the aging process [137].

Both intrinsic and extrinsic aging cause significant degradation of HA, leading to a range of clinical signs of skin aging [7,111]. These symptoms include excessive dryness, the formation of wrinkles, and the loss of the firmness and elasticity of the skin [7,111]. In the case of chronologically aged skin, a reduced amount of HA-binding protein (HABP) has been observed in the dermis. HABP is a protease that, through the mitogen-activated protein kinase (MAPK) ERK1/2 and Pi3K/Akt signaling pathways, promotes the migration and proliferation of fibroblasts [134,138]. However, the level of HA does not undergo drastic changes [7]. Interestingly, the most significant loss of HA occurs in the epidermis, while the amount in the dermis does not change significantly [7]. It has been found that the loss of HA in the process of intrinsic aging is also associated with a decrease in the activity of HAS1 and HAS2 as well as CD44 and RHAMM [7]. It is believed that the decrease in HA synthesis during aging may also be a result of elevated levels of non-coding molecules like miR23-3p. It has been shown that miR23-3p binds to the untranslated region (3’UTR) of mRNA located downstream of the coding sequence, which leads to a reduction in the expression of HAS2 and, consequently, a decrease in fibroblast proliferation and HA synthesis [139].

Although photoaging is known to be responsible for premature skin aging, the research results regarding changes in HA metabolism in photo-damaged skin are inconclusive [15,39]. Relatively few studies have been conducted on healthy volunteers regarding the role of HA in photoaging induced by UV radiation. Averbeck et al. have demonstrated that in skin exposed to UVB radiation, the level of HA increases in the epidermis, while the amount of HA in the dermis decreases due to the reduced expression of HAS1 and HAS2 in this layer [15]. Interestingly, a statistically significant increase in the concentration of HA fragments with a molecular weight <100 kDa was observed in the interstitial fluid of the dermis 24 h after UVB exposure (95.85 ± 40.09 ng/mL) compared to the control group (4.809 ± 1.264 ng/mL) [140]. Another in vivo study has shown that UVB radiation increases the concentration of chondroitin sulfate in the dermis but not HA itself [15]. Importantly, it has been observed that in skin exposed to UV radiation, where damage to the basement membrane occurs, the amount of HA in the epidermis is reduced compared to that in skin protected from the sun and young skin exposed to sunlight with an intact basement membrane [15]. It has been hypothesized that the basement membrane maintains HA levels in the epidermis [15]. Most authors agree that when skin is exposed to UV radiation, there is a decrease in the activity of HAS1 and HAS2. Changes in the activity of hyaluronidase can also be observed. In UV-exposed skin, the levels of hyaluronidase-1, hyaluronidase-2, hyaluronidase-3, and the hyaluronan-binding protein (CEMIP) increase. These changes are suspected to lead to an increase in the catabolism of HA [15]. In vivo studies on mouse models have shown that in skin exposed to UVB radiation, the concentration of HA is lower in the papillary dermis compared to that in skin protected from the sun [15,141]. Interestingly, LMW-HA is mainly present in skin exposed to UV light [15,141]. Additionally, HA produced during photoaging (LMW-HA) cannot bind enough water [15]. Indeed, HMW-HA has a higher hydration potential than LMW-HA. HMW-HA’s water-binding mechanism is related to the ability of this molecule to form numerous hydrogen bonds with water, which is also influenced by the presence of numerous polar groups such as hydroxyl (-OH), carboxyl (-COOH), and acetamide (-NHCOCH_3_). Molecular dynamic simulations have shown that the number of hydrogen bonds possible increases with the length of the polymer chain. Additionally, HMW-HA chains show the ability to self-organize into a three-dimensional supramolecular network stabilized by non-covalent bonds, increasing the ability to bind water [142].

### 3.3. Hyaluronic Acid in Anti-Aging Prevention

**Figure 1:**
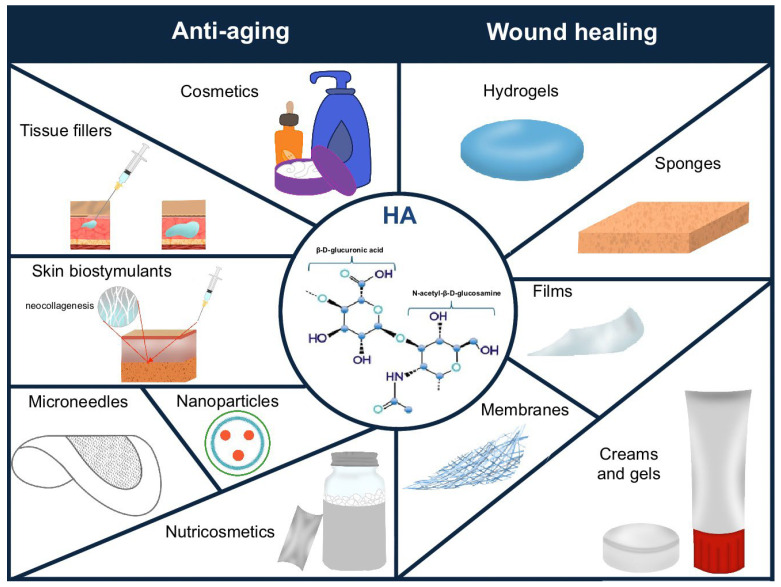
The use of hyaluronic acid (HA) in aging prevention and wound healing.

HA is widely used in clinical medicine, aesthetic medicine, and cosmetology [51]. Numerous studies have confirmed the safety and effectiveness of HA in improving skin hydration and reducing visible signs of aging [14,134,143,144]. HA is one of the most popular ingredients in cosmetic products and tissue fillers, commonly used for restoring volume lost over the course of aging [145]. However, many researchers are increasingly emphasizing the beneficial role of HA supplementation in improving skin quality and appearance [4,51]. The uses of hyaluronic acid in aesthetic medicine and cosmetology are shown in Figure 1.

#### 3.3.1. Anti-Aging Products

Currently, HA is widely used in the cosmetics industry [146,147]. HA’s rheological, viscoelastic, and hygroscopic properties have made it a key active ingredient in many cosmetics [146,147]. Sodium and potassium hyaluronate are widely used in moisturizing products intended for facial and body care as well as in eye-area treatments and anti-wrinkle cosmetics [14,144]. HA is commonly used in creams, gels, serums, masks, and lotions [14,144]. In cosmetic formulations, HA may be used as a standalone ingredient or in combination with excipients that facilitate its penetration into deeper layers of the skin, such as liposomes [14,134,143]. HA in cosmetic products is often combined with other bioactive ingredients, particularly amino acids, peptides, vitamins, plant extracts, and probiotics, to enhance its effect or provide additional benefits for the skin [14]. The concentration of HA in cosmetics typically ranges from 0.2% to 1% [14]. HA’s different biological effects in cosmetic products depend on its molecular weight [14,148]. High-molecular-weight HA has demonstrated the ability to form an occlusive film on the skin’s surface, preventing transepidermal water loss (TEWL) in the epidermis and protecting against the adverse effects of external factors while also contributing to the maintenance of proper hydration levels [14]. However, HMW-HA does not penetrate the deeper layers of the skin. LMW-HA exhibits better permeability to both the epidermis and the dermis [148]. Therefore, iontophoresis, electroporation, and microneedles can improve HA’s penetration through the stratum corneum. Iontophoresis increases the diffusion of therapeutic substances/active ingredients through the skin [149]. This method involves the use of a low-voltage direct current, which is necessary to move charged particles through the skin. Inoue et al. evaluated the effectiveness of iontophoresis in delivering HMW-HA with a molecular weight of 600–1600 kDa [150]. They showed that iontophoresis enabled HMW-HA to penetrate the skin [150]. Fluorescent labeling showed the presence of HMW-HA in the epidermis (up to 100 μm) and in the dermis (over 200 μm). There was no significant fluorescence in the control group, where HMW-HA was applied to the surface of skin without iontophoresis, indicating that HMW-HA does not cross the epidermal barrier on its own [150]. Electroporation is another method that allows active substances to penetrate the skin. It involves the use of brief electrical impulses to create temporary pores in the cell membrane, which facilitate the transport of a variety of substances into the skin [151]. HA penetration was shown to be greater with a combination of electroporation and iontophoresis compared to iontophoresis alone. It was observed that the maximum molecular weight of HA that was able to penetrate the skin was 200 kDa [152]. Cosmetic products can also contain combinations of HA with different molecular weights, balancing HA’s penetration and biological activity [50].

The mechanisms of action and penetration of HA through the skin, depending on its molecular weight, are presented in Table 2. HA also plays a valuable role in anti-aging [14]. It delays the skin-aging process, reduces wrinkles, and prevents the formation of new wrinkles by improving water retention in the skin, limiting water loss, smoothing the texture of the skin, and strengthening the skin’s structure [14]. Clinical studies have evaluated the effectiveness of topical HA preparations with different molecular weights (50, 130, 300, 800, and 2000 kDa). The use of HA with molecular weights of 50 and 130 kDa was shown to have contributed to a significant reduction in wrinkles, which may have been due to the ability of low-molecular-weight HA to penetrate the deeper layers of the epidermis [153].

#### 3.3.2. Tissue Fillers

The use of aesthetic medicinal procedures is gaining increasing interest in regard to anti-aging therapies [161]. A particularly sought-after procedure among minimally invasive treatments is the injection of tissue fillers. HA-based fillers are clinically indicated for soft tissue augmentation, nasolabial fold and wrinkle correction, and lip enhancement, including volume restoration and vermilion border contouring [144,161]. These preparations are characterized by good water-binding properties that help increase the volume of soft tissues [162]. Crosslinked HA is the most popular and effective ingredient in tissue fillers [161]. Non-cross-linked HA has a short half-life, usually ranging from 1 to 2 days [10,163]. When dissolved in water, HA forms a sticky solution that does not fulfill the role of a skin filler [10,163]. It quickly degrades by hyaluronidase and does not stay in the injection site [164]. In order to extend durability and improve elasticity, various HA production technologies and crosslinking methods are used [10,163]. Products containing crosslinked HA may be classified into monophasic or biphasic, depending on the method of crosslinking [162]. Monophasic fillers are based on a homogeneous gel of crosslinked HA, which ensures greater stability at the injection site and reduces the susceptibility of HA to enzymatic breakdown [155]. Biphasic products consist of stabilized HA molecules in the form of a gel dispersed in a non-crosslinked HA solution [162]. The effects of using HA fillers usually last from 6 to 12 months after injection [10]. Recent studies indicate that HA fillers, depending on the type of product used, can last longer, even up to 15 years [165]. However, further studies are required to confirm the long-term effects of HA. Tissue fillers based on HA have a high safety profile [166]. However, side effects may occur after use, as with all products in cosmetology and aesthetic medicine [166]. However, they are usually short-term, mild, and transient [166,167]. The main complications associated with the injection of tissue fillers containing HA include pain, swelling, itching, bruising, and hypersensitivity reactions [166,167]. These side effects are often due to an immune response caused by the contaminants present in HA formulations, such as proteins, nucleic acids, bacterial endotoxins, or heavy metals [168,169]. Purified HA or HA produced by recombinant bacteria is used to minimize the severity of allergic or unwanted immune reactions [168,169]. Ultrafiltration and diafiltration are particularly preferred methods for purifying HA due to their selectivity and efficiency [169]. Ultrafiltration involves the use of semipermeable membranes that retain large particles and allow smaller ones to pass through [169]. After ultrafiltration, diafiltration is used to remove contaminants by exchanging the solvent and gradually rinsing out unwanted substances, thus yielding high-purity HA [169]. Other methods of purifying HA include centrifugation, precipitation, and chromatographic techniques (size-exclusion chromatography and high-pressure liquid chromatography) [169]. HA is also produced using biotechnological methods in which genetically modified microorganisms, such as *Escherichia coli* and *Bacillus subtilis*, with a status recognized as safe (GRAS) synthesize HA in a controlled environment [21,51]. Compared to traditional methods, HA produced by recombinant microorganisms poses a reduced risk of contamination [21,51]. For polymeric materials such as HA, it is necessary to determine the characteristic parameters according to the Pharmacopoeia, American Society for Testing and Materials (ASTM), or International Organization for Standardization (ISO). Each product should have a specification with approved analytical methods and acceptance criteria to confirm its safety before clinical use [170]. Chung et al. have proposed a skin test for HA-containing products to minimize allergic reactions [171]. However, the time required to interpret the results can be as long as 3–4 weeks. This skin test is particularly recommended for patients who have previously experienced adverse reactions to HA fillers. In case of a positive result, the patient should not undergo treatment with the same preparation again. However, it should be noted that the skin test does not entirely eliminate the risk of adverse reactions, which can be caused by concomitant factors such as infection [171].

In rare situations, hazardous complications related to blood vessels may occur, which may pose a threat to health and life [166]. Among these complications, skin necrosis, ischemic stroke, and vision loss are distinguished as the severe ones [166,167]. Vascular complications after the use of HA-based dermal fillers result from vascular compression due to pressure placed on the vessel by the filler material [151]. Impaired blood flow is the result of vessel occlusion [167]. Vascular complications are characterized by a rapid course, leading to irreversible changes [167]. Therefore, an operator with a thorough knowledge of anatomy, early recognition of the signs and symptoms of vascular complications, and immediate implementation of treatment may help avoid dangerous health consequences [167].

##### Cross-Linking of Hyaluronic Acid

Various HA crosslinking methods, including chemical, physical, and enzymatic methods, are used to extend HA-based hydrogels’ durability and improve their elasticity and ability to resist enzymatic degradation [10,163].

HA chemical cross-linking involves the use of various chemicals, such as 1,4-butanediol diglycidyl ether (BDDE), divinyl sulfone (DVS), glutaraldehyde (GTA), and 1-ethyl-3-(3-dimethylaminopropyl carbodiimide/N-hydroxysuccinimide (EDC/NHS) [172,173]. HA’s most common crosslinking agent is BDDE [10,162,163]. The reaction of BDDE epoxide groups with HA hydroxyl groups in an alkaline environment forms ether bonds that crosslink HA molecules through 1,4-butylene bridges, forming a three-dimensional structure [174]. It has been shown that ether bonds ensure there is significant clinical durability while maintaining biodegradability [175,176]. Unreacted BDDE is non-toxic when its concentration in the end product does not exceed 2 ppm [177]. BDDE is widely used in tissue fillers and injectable hydrogels [176]. DVS is a less commonly used chemical cross-linking agent [10,162,163]. DVS reacts at room temperature, thereby minimizing HA’s degradation in alkaline solutions compared to methods requiring elevated temperatures [172]. DVS’s high reactivity leads to the formation of ether bonds in a reproducible process without using organic solvents [172]. Unlike BDDE, DVS is highly toxic [163]. GTA was once a common crosslinking agent due to its ability to increase the stability and effectiveness of crosslinking [173,178]. However, studies have shown that it has significant toxicity, and residual GTA can reduce the biocompatibility of gels [173]. Another HA cross-linking agent is EDC/NHS. EDC/NHS is crucial for forming amide bonds between HA’s carboxyl groups and other molecules’ amino groups [179]. The EDC/NHS cross-linking method has been used in tissue engineering and the creation of drug carriers [179].

In contrast to chemical cross-linking, the physical cross-linking of HA hydrogels involves the use of non-covalent interactions, such as hydrogen bonds, electrostatic interactions, and the entanglement of the polymer chain, to form a three-dimensional network [172,180]. Physical stimuli modulate these reversible interactions: temperature, pH, and ionic bonds [172,180]. Physically cross-linked HA hydrogels are usually characterized by high biocompatibility and are used in various biomedical applications, particularly in tissue engineering, drug delivery systems, and wound healing [172,180].

Enzymatic crosslinking involves the use of enzymes, mainly horseradish peroxidase (HRP), to form covalent bonds between HA molecules [172]. Although enzymatic crosslinking is a highly biocompatible method, it can lead to variations in gelling kinetics between different batches of material [172]. Enzymatically cross-linked HA hydrogels are used in wound healing, tissue engineering, and drug delivery systems [172].

#### 3.3.3. Skin Biostimulants

The dynamic development of aesthetic medicine has contributed to the development of new methods used in anti-aging medicine [181]. One of the most popular anti-aging treatments is skin stimulation using biostimulants [181,182]. In contrast to tissue fillers, the role of skin biostimulants is not to increase tissue volume and fill wrinkles but to naturally rebuild and regenerate aging skin [181,182]. The action of skin biostimulants involves stimulating fibroblast activity to produce collagen and elastin [181]. The effects of the procedure are not immediate and usually become visible gradually after several treatments [182]. The frequency of using skin biostimulants varies and depends on the type of product used, the condition of the patient’s skin, and the aesthetic goal [183]. A particular advantage of these products is their ability to yield natural results in terms of improved hydration, firmness, and skin thickening without altering facial features [182]. A common ingredient in tissue stimulators is non-crosslinked HA combined with other substances such as polynucleotides (Mastelli Nevest^®^, Milano, Italy); amino acids, including glycine, L-proline, L-lysine, L-valine, and L-leucine (Sunekos 200^®^, Milano, Italy); L-polylactic acid (Juvelook^®^, Seoul, Republic of Korea); and 1% calcium hydroxyapatite (Neuvia Stimulate^®^, Geneve, Switzerland) [183,184,185,186,187,188,189]. Few studies have evaluated HA’s effectiveness as an agent stimulating neocollagenesis [184,190]. However, a recent study has shown that the use of non-crosslinked HA in a skin biostimulator exhibited stimulating effects [184]. Optical coherence tomography was used to assess collagen density, allowing for the examination of skin layers up to a depth of 2 mm. Optical coherence tomography imaging was performed before skin stimulator injection and at 1, 2, and 3 months post-injection. A progressive increase in collagen density was observed, even up to 3 months after a single injection [184]. Further studies are necessary to confirm the effectiveness of HA in skin biostimulation [190].

#### 3.3.4. Microneedles

Microneedles (MNs) constitute an innovative transdermal system that allows the delivery of hydrophilic (such as HA) and hydrophobic substances by mechanically disrupting the stratum corneum. MNs are small structures ranging from 25 μm to 2000 μm. They have diverse shapes, and they can be made of different materials (metal, ceramics, silicon, and biodegradable polymers) [191,192,193]. Depending on their mechanisms of action, structures, and materials, MNs are divided into solid, hollow, coated, hydrogel, and soluble MNs [191,193]. MNs have a wide range of applications, ranging from the transdermal administration of pharmaceuticals such as vaccines, proteins, and immunological drugs to diagnostic and cosmetic applications [194,195]. Studies have shown that composite MNs with nanocarriers increase the effectiveness of drug loading and improve therapeutic efficacy [196]. This technology is a promising alternative to traditional methods such as injections or topical preparations, providing a non-invasive and effective route for transdermal drug delivery [195].

HA is a very common material used for the production of soluble MNs due to its properties, such as biodegradability and biocompatibility [196]. Soluble HA MNs dissolve in the skin. Therefore, they do not generate sharp waste, reducing the risk of infection [196]. While hyaluronic acid (HA) is a natural carboxyl group-containing polymer, the hyaluronic acid-based microneedle patch (HA-MNP) system provides a transient acidic microenvironment with oxygen reduction at the administration site. These conditions inhibit the degradation and dimerization of many drugs and active substances [191]. In addition, HA MNs have the ability to encapsulate and stabilize sensitive biological particles [191]. HA MNs can be modified by adding other active substances to them, such as peptides, to improve skin condition [193]. HA MNs are also used in dermatology to aid in the treatment of diseases such as psoriasis and melasma and to promote wound healing [193]. HA MNs are also being researched for their potential as drug delivery systems in anti-cancer therapy [193]. Several methods for preparing soluble HA MNs include micro-molding, photopolymerization, drawing lithography, and, most commonly, solvent casting [196]. Lu et al. used HA MNs as a system for the controlled release of a therapeutic complex consisting of lipoic acid (LA) and the self-organizing peptide RADA16-YIGSR (RY) in the treatment of diabetic wounds [197]. The HA (RY/LA) MN system led to increased collagen synthesis after 14 days (67.25%), which can be compared to the value of 25.84% in the control group, indicating faster wound healing. HA (RY/LA) MNs also reduced the survival rate of *Escherichia coli* to 10.2% and that of *Staphylococcus aureus* to 9.1%. After 48 h of HA (RY/LA) MN application, a 2-fold increase in endothelial cell migration (HUVEC) and the formation of numerous tubular structures were observed, indicating proangiogenic effects [197].

#### 3.3.5. HA-Based Nanoparticles

Nanotechnology, one of the fastest-growing fields of science, is widely used in cosmetology, pharmacy, and medicine [198]. Lipid-based nanoformulations such as liposomes, niosomes, ethosomes, polymeric micelles, solid lipid nanoparticles, nanostructured lipid carriers, and nanoemulsions serve as carriers for therapeutic substances [46,198]. They enable controlled drug release, action targeted at a specific destination, and increased penetration into the skin. Many studies have shown that HA-coated nanoparticles are highly effective in delivering poorly water-soluble drugs and ensuring their controlled release [46,199,200].

Liposomes are vesicles composed of phospholipid bilayers, the size of which can range from 30 nm to several micrometers. Liposomes have unique properties as drug carriers, making them widely used in pharmacy and medicine [198,201,202]. Liposomes are biocompatible and protect the substances they contain from physiological degradation, thus extending their half-lives and allowing for controlled release [201,202,203]. In cosmetics, liposomes are most often used as carriers of fragrances in deodorants, antiperspirants, and various moisturizing products [204,205]. Chang et al. evaluated the effectiveness of HA-modified cationic liposomes in enhancing skin penetration and retention. They found that LMW-HA with molecular weights of 5 kDa and 8 kDa could penetrate the dermis. Cationic liposomes containing undecylenoylphenylalanine coated with 5 kDa HA showed 5.4-times greater penetration and 4.9-times greater retention in the skin compared to undecylenoylphenylalanine alone, which is a skin-brightening agent [143]. HA-modified liposomes also significantly advance targeted drug delivery systems [46,202,206]. Coating liposomes with HA significantly increases the effectiveness of drug encapsulation. Due to the affinity of HA for CD44 receptors, which are overexpressed in some cancer cells, HA-modified liposomes enable a drug to be delivered directly to the tumor, thus increasing therapeutic efficacy [46,206].

Ethosomes are lipid carriers consisting of phospholipids and ethanol or isopropanol, usually in a concentration of 20–50% [207,208]. Compared to liposomes, they are more effective at penetrating the epidermal barrier, which allows them to deliver drugs to deeper layers of the skin [207,209,210]. In pharmacy, they are used to deliver antibiotics, painkillers, hormones, or anti-inflammatory drugs [207,210]. Ethosomes are also commonly used in cosmetics as carriers of active ingredients such as HA. The encapsulation of HA in ethosomes increases its biodegradability and protects it from enzymatic degradation [211,212].

Niosomes are small vesicles made of non-ionic surfactants. The bilayer structures of niosomes allow the encapsulation of different compounds, i.e., amphiphilic, hydrophilic, and lipophilic compounds [213,214]. Niosomes are more stable and bioavailable and less toxic compared to other carriers. Hanieh et al. prepared HA- and cholesterol-coated (HA-Chol) niosomes. They showed that the HA-Chol coating improved the niosomes’ stability, allowing for controlled release and selective delivery of drugs to specific cells, particularly cancer cells [215].

Polymeric micelles integrating HA are self-organizing nanoparticles with a core–shell structure. They encapsulate hydrophobic drugs in their cores, while their hydrophilic HA shells ensure water solubility and colloidal stability [216,217]. Šmejkalová et al. evaluated the effectiveness of polymeric micelles integrating hydrophobized HA in the local delivery of drugs through the skin [218]. The polymeric micelles integrating HA led to a 3-fold higher drug concentration in the epidermis and a 6-fold higher concentration in the dermis than conventional non-micellar systems [218].

Solid lipid nanoparticles (SLNs) are composed of solid lipids, usually between 50 and 1000 nm in size [219]. The lipid core surrounds the drug, ensuring its controlled release and protection against degradation. The modification of solid lipid particles with HA (HA-SLN) improves their ability to target specific cells [46,219,220]. This is due to the affinity of HA for CD44 and RHAMM receptors, which are overexpressed in tumor and inflammatory cells [46,219].

Nanostructured HA-modified lipid carriers (HA-NLCs) consist of solid and liquid lipids. This hybrid structure allows for a greater drug-loading capacity and better release control compared to conventional carriers [46,221].

Nanoemulsions are colloidal dispersion systems of the oil-in-water (o/w) or water-in-oil (w/o) type. The small size of nanoemulsions, in the 20–200 nm range, facilitates their effectiveness as drug carriers [222,223]. Nanoemulsions are used in many cosmetic formulations, including body lotions, sunscreens, anti-aging, and whitening preparations. They help rebuild the hydrolipid barrier, making them particularly suitable for dry-skin care [204,224]. Kong et al. evaluated the skin penetration, safety, and bioavailability of nanoemulsions containing HA as a carrier for lipophilic α-tocopherol. It was shown that HA nanoemulsions are safe and have better permeability through the stratum corneum compared to ethanol [225].

#### 3.3.6. Nutricosmetics

Although HA is primarily an ingredient in topical products, it is also a common component in nutricosmetics [144]. Nutricosmetics constitute a subcategory of dietary supplements intended to improve the appearance of the skin, hair, and nails [226]. The main purpose of nutricosmetics is to supplement deficiencies in nutrients that may cause excessive dryness and roughness of the skin, loss of elasticity, and premature skin aging [226]. Nutricosmetics have various effects depending on the type of nutrients they contain. They may improve skin hydration, act in a antioxidative capacity, strengthen the skin’s structural integrity, and exhibit photoprotective properties [226,227]. Supplements with HA are available in the form of tablets, capsules, powder, or liquid [227]. Regarding nutricosmetics, HA is most often combined with other essential skin ingredients such as collagen, vitamins, minerals, and plant extracts [14,226]. According to the related literature, HA supplementation improves quality and elasticity, increases skin hydration, and reduces the expression of wrinkles [227]. It has been shown that different doses and molecular weights of HA benefit the skin, and the effectiveness of nutricosmetics primarily depends on regular use [227]. However, further studies are required to confirm the effectiveness of HA in oral supplementation and determine the optimal dose and form of HA [14].

## 4. Hyaluronic Acid in Wound Healing

Wound healing is a multifaceted physiological process, the duration of which depends on the type and size of the injury [8]. Wound healing begins with hemostasis, during which blood clotting and fibrinolysis occur [54,228,229,230]. Blood clotting is initiated through two pathways: the extrinsic and intrinsic pathways. The extrinsic pathway is triggered by tissue factor (TF) that is released from the damaged blood vessel. TF then binds to factor VIIa (proconvertin) and activates factor X (Stuart–Prower factor). In the intrinsic pathway, factor XII (Hageman factor) is activated, which subsequently activates factor XI (antihemophilic factor C). This activation then stimulates factor IX (antihemophilic factor B) and factor VIIIa (antihemophilic factor A), further stimulating factor X. Both pathways converge at the activation of factor X and factor Xa, forming the common pathway. As a result of factor Xa activation and factor Va (proaccelerin), prothrombin is converted into thrombin. Thrombin plays a key role in hemostasis, particularly in converting fibrinogen into fibrin, leading to clot formation [228,229,230,231]. Fibrinolysis is the process whereby blood clots dissolve in blood vessels. It is initiated by tissue plasminogen activator (tPA) and urokinase plasminogen activator (uPA), which convert plasminogen into plasmin, leading to the degradation of fibrin [232].

Next, the inflammatory phase occurs, which is crucial in wound healing. During the inflammatory phase, the immune system is activated. Neutrophils accumulate at the wound site, recognizing and phagocytizing pathogens, while macrophages promote tissue repair [233]. The next stage is the migration and proliferation of fibroblasts to the wound site. During this phase, the tissue defects are filled with granulation tissue rich in blood vessels, fibroblasts, and collagen fibers. The final stage consists of remodeling and scar formation [88].

Many studies have shown that HA actively participates in all the stages of wound healing. The focus has primarily been on the impact of HA on the process of angiogenesis [55]. The interaction of HA with CD44 and RHAMM receptors, as well as with immune system cells, induces the production of many compounds crucial for the healing process [55,234]. Among these compounds, cytokines are crucial, including TGF-β, transforming growth factor alpha (TGF-α), basic fibroblast growth factor (bFGF), epidermal growth factor (EGF), TNF-α, IL-1β, IL-8, and many others initiated during the inflammatory phase [234]. TLR2 and TLR4, as well as lymphatic vessel endothelial hyaluronan receptor-1, bind to HA and play a significant role in the healing process. The interaction between HA and TLRs causes LMW HA to exhibit proinflammatory properties [26,234,235].

In the hemostasis phase, HMW-HA accumulates in the wound bed and then binds to fibrin and fibronectin, forming a temporary scaffold that facilitates the accumulation of fibroblasts and inflammatory cells such as lymphocytes, neutrophils, and macrophages [62].

The amount of HA synthesized significantly increases during the inflammatory phase [88]. The unique properties of HA, especially its hydrophilicity, allow for the passive diffusion of water into the interstitial space. As a result, edema forms around the wound, and chemotaxis of inflammatory cells occurs. Fragments of HA with low or medium molecular weights activate the synthesis of proinflammatory cytokines, TNF-α, IL-1β, and IL-8, which influence the dilation of blood vessels, leading to redness of the skin and increased warmth at the site of the injury [62,88]. A key role in inflammation is also played by the HA-binding protein known as TNF-stimulated gene 6 (TSG-6) and the serine protease inhibitor (IαI). As a result of the interaction between the TSG-6/IαI complex and HA, the inflammatory phase is attenuated through a cascade of proteases and MMPs, which leads to the migration of neutrophils and the inhibition of plasmin, an active component of the fibrinolytic system [62,88,236]. The inflammatory phase and the associated symptoms are essential elements in the proper regeneration of the skin and wound healing [88].

HA also plays an important role in the proliferative phase. During this stage, fibroblasts migrate to the site of the wound. This process is facilitated by small fragments of HA consisting of 6–20 saccharides and growth factors [26,62,88]. Subsequently, fibroblasts produce glycosaminoglycans and collagen that facilitate ECM remodeling [88]. HA also significantly influences neovascularization [11]. The involvement of HA in angiogenesis depends on its molecular weight [51]. HMW-HA inhibits angiogenesis, whereas LMW-HA exhibits proangiogenic effects [55]. Additionally, LMW-HA supports the epidermalization phase. This is related to the interaction of CD44 in endothelial cells with HA oligomers. Activated endothelial cells stimulate the processes of migration, granulation, and the formation of new blood vessels [237].

In the remodeling phase, as a result of fibroblast activity, the edges of a wound contract [62]. In this phase, HA also plays an important role. Primarily, it integrates with CD44. The activation of CD44 stimulates cells derived from mesenchyme, referred to as myofibroblasts. Myofibroblasts are characterized by their ability to move and their capacity to produce ECM components [237,238]. They constitute one of the key elements that condition a proper wound-healing process. Primarily, they facilitate the closure of a wound [12]. Moreover, they are responsible for the production of MWM-HA, most often with a molecular weight of approximately 480 kDa [235]. LMW-HA enhances the expression of TGF-β1 and TGF-β2, which are responsible for scar formation. On the other hand, HMW-HA increases the expression of TGF-β3, which is involved in inhibiting the scarring process [61].

### 4.1. Preparations Containing Hyaluronic Acid Used in Wound Treatment

**Figure 2:**
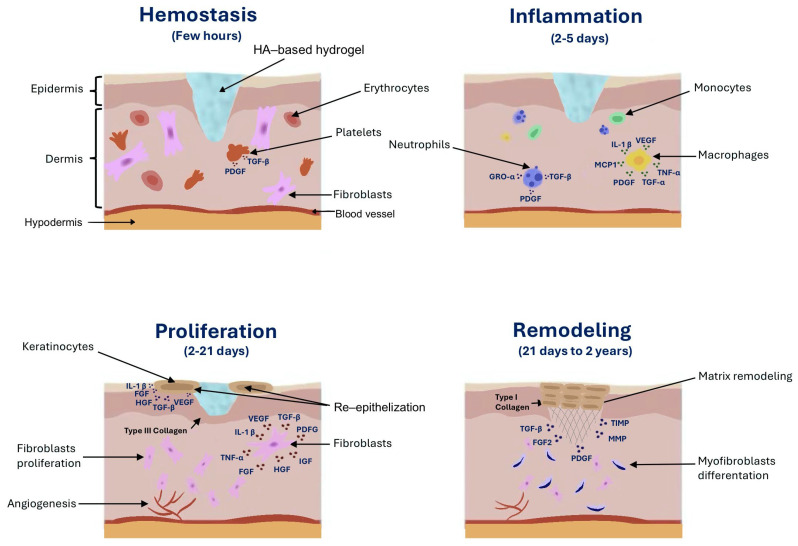
The role of hyaluronic-acid-based hydrogel in wound healing.

Due to its strong tissue dehydration prevention properties, effectiveness in supporting and accelerating the regeneration of damaged skin structures, and biocompatibility and low cytotoxicity, HA is increasingly used as an ingredient in wound-healing preparations. HA is used in various types of dressings, such as hydrogels, sponges, films, and electrospun membranes, as well as in creams and foams [26,239] (Figure 1). HA is used in wound dressings to maintain a moist environment within the damaged tissue, reduce the production of proinflammatory cytokines (↑TNF-α, ↑IL-1β, and ↑monocyte chemoattractant protein-1 (MCP-1)), intensify the expression of growth factors (↑TGF-β, ↑platelet-derived growth factor (PDGF), ↑growth-regulated oncogene alpha (GRO-α), ↑vascular endothelial growth factor (VEGF), ↑fibroblast growth factor (FGF), ↑hepatocyte growth factor (HGF), ↑insulin-like growth factor (IGF), and ↑fibroblast growth factor 2 (FGF2)), stimulate cell migration and proliferation (↑TGF-α), improve granulation, and enhance the formation of blood vessels [237,240] (Figure 2). Many clinical studies have confirmed the positive effects of using various types of HA dressings in wound healing, particularly for postoperative wounds and chronic ulcers. They are more effective but also more expensive than standard wound treatment strategies, limiting their widespread use [241]. In order to reduce production costs, it is important to increase fermentation efficiency, which can be achieved by optimizing breeding conditions and using genetically modified microorganisms [242]. Ferreira et al. analyzed the economics of HA production via *Streptococcus zooepidemicus* fermentation [242]. They compared batch fermentation and fermentation with feed-batch substrate dosing [242]. It was proven that feed-batch fermentation leads to a higher HA concentration (5.0 g/L) compared to the batch method (2.5 g/L). The authors also showed that the use of feed-batch fermentation reduced production costs (USD 946/kg) compared to batch fermentation (USD 1115/kg) [242].

IL-1β: Interleukin-1 beta; FGF: fibroblast growth factor; FGF2: fibroblast growth factor 2; GRO-α: growth-regulated oncogene alpha; HGF: hepatocyte growth factor; IGF: insulin-like growth factor; MCP-1: monocyte chemoattractant protein-1; MMPs: matrix metalloproteinases; PDGF: platelet-derived growth factor; TGF-β: transforming growth factor beta; TIMP: tissue inhibitor of metalloproteinase; TGF-α: transforming growth factor alpha; TNF- α: tumor necrosis factor alpha; VEGF: vascular endothelial growth factor.

#### 4.1.1. Hydrogels

HA is one of the common ingredients in hydrogel dressings. HA-based hydrogels are three-dimensional polymer networks consisting of randomly linked HA chains. Compared to natural ECM, HA-based hydrogels are characterized by lower structural complexity and limited functional diversity [243]. Hydrogel dressings are widely used in wound healing due to their hydrophilicity, biodegradability, and cytocompatibility [243,244]. In hydrogel dressings, HA facilitates tissue regeneration by accelerating the stages of wound healing. Additionally, HA reduces inflammation during the inflammatory phase, stimulates angiogenesis during the proliferative phase, and enhances collagen deposition during the remodeling phase [244]. HA is most commonly used in hydrogel dressings for treating chronic wounds, such as ulcers or pressure sores [245]. However, there are certain limitations associated with the use of HA-based hydrogels. These include the poor mechanical strength of the resultant gels and the rapid degradation of HA [239]. Thus, to accelerate the healing process, the HA in hydrogel dressings is often combined with EGF or other biomaterials. EGF also promotes tissue regeneration through cell stimulation and proliferation [246]. Chemical modifications and crosslinking with synthetic polymers are also used in HA-based hydrogels, significantly helping to overcome the mentioned limitations [239]. Gong et al. were the first to propose a new hydrogel class combining one rigid and strong network with another soft and elastic one, forming dual-crosslinked hydrogels. In this type of system, two independent networks formed through different types of chemical and physical crosslinking coexist within a single hydrogel, maintaining structural separation [247]. The structural integrity of hydrogels can be increased through crosslinking with synthetic polymers such as polyacrylamide (PA) and polyvinyl alcohol (PVA) [248]. The synthetic polymers mixed into the hydrogel form intermolecular bonds that strengthen the structure of the material [248]. PA increases the mechanical strength of hydrogels due to its ability to form strong covalent bonds [248,249]. PVA, on the other hand, forms hydrogen bonds that can make hydrogel flexible [248,250]. In order to inhibit the enzymatic degradation of HA, Lee et al. proposed adding tannic acid and polyethylene glycol (PEG) diglycidyl ether to crosslinked HA hydrogels. Due to its hydroxyl groups, tannic acid forms additional hydrogen bonds with the diglycidyl ether of PEG, which contributes to the enhancement of the physicochemical properties of a hydrogel [251].

The interactions and kinetics of HA release in polymer hydrogels constitute a significant challenge that requires further research and optimization. An uncontrolled or excessively rapid release of active substances can decrease therapeutic efficacy [3]. The use of gradient polymer systems can be an effective approach to regulating the diffusion of medicinal substances in HA-based hydrogels. Gradient hydrogels are characterized by their anisotropic structures, and their properties vary spatially [252]. In the case of gradient hydrogels, the release kinetics can be optimized by controlling the differences in the drug concentration in the polymer matrix. This enables a more even release of the active substance in the later stages [253]. One of the most promising solutions for controlling the release of substances is the use of stimuli-responsive hydrogels. Intelligent hydrogels react to factors such as pH, light, temperature, or enzymes and glucose [254]. Physical crosslinking is often preferred due to the reversibility of supramolecular interactions [255].

#### 4.1.2. HA-Based Sponges

For acute and chronic wounds, HA-based sponges (HylaSponge^®^) may be used [246]. The sponges’ porous structures and swelling ability allow maintaining a moist environment within the wound and absorbing excess exudate. In order to keep the sponges in place on a wound, additional dressings or bandages are required [256]. HA is also combined with other polymers, such as chitosan, to improve the mechanical properties of HA-based sponges [257]. Chitosan is biocompatible and has an antimicrobial effect. Chitosan-HA composites constitute a material with better mechanical properties [257]. Mohandas et al. created VEGF-coated fibrin nanoparticles and introduced them into a chitosan-HA composite sponge intended as a dressing for diabetic wounds [257]. The related literature data indicate that the use of HA sponges with a zinc system significantly reduces scarring in patients after breast surgery [245,258,259]. HA-based sponges combined with chitosan and nanosilver have shown effectiveness in treating diabetic foot ulcers among patients infected with antibiotic-resistant bacteria [257]. Catanzano et al. developed a composite alginate–HA sponge dressing loaded with tranexamic acid [260]. Alginate has hemostatic properties. Therefore, HA-and-alginate sponges can effectively heal wounds and reduce bleeding after tooth extractions [260]. HA-and-aloe-based sponges are beneficial in the case of burns and diabetic ulcers [261]. Foam dressings are used for chronic wounds with moderate or heavy exudate [262]. They do not adhere to the wound bed and support epithelialization [262,263]. Kim et al. modified HA dressings by introducing methacrylic acid (MAA) groups [264]. It has been shown that MAA-containing HA dressings (HAMA) accelerate the wound-healing process [264]. These dressings ensure high absorption of exudate [264].

#### 4.1.3. Hyaluronate-Based Dressings

Hyaff-11^®^ is a technology primarily developed for tissue-engineering purposes [265]. By esterifying the free carboxyl group of glucuronic acid with benzyl alcohol, a polymer of the HA derivative, called benzyl ester of HA, was obtained [266]. A unique feature of this polymer is its biodegradability and improved stability compared to native HA, all while retaining HA’s biological properties [265,267]. In a clinical study involving 180 participants, the effectiveness of autologous skin grafts cultured on scaffolds made of benzyl ester HA (Hyaff) was evaluated with respect to the treatment of diabetic foot ulcers. Nonadherent paraffin gauze was used in the control group (90 participants). In the treatment group (90 participants), an autologous skin graft was applied using tissue-engineering methods (Hyalograft-3D), and after 2 weeks, an epidermal autograft (Laserskin) was applied. After 12 weeks, it was found that the healing process progressed similarly in both groups. However, in the treatment group, the reduction in the ulcer area occurred faster (40 days) than in the control group (50 days). After 20 weeks, 50% of the treatment group achieved ulcer healing, while only 43% of the control group had healed ulcers. Further studies are needed to confirm the effectiveness of Hyaff scaffolds for patients with ulcers at other locations [268].

Transparent HA films (Hyalosafe^®^) are another type of dressing using Hyaff-11. They are used in the treatment of superficial wounds with moderate exudate as well as for first- and second-degree burns. These HA films are designed to create a moist environment necessary for healing and provide a physical barrier to protect a wound from infection [245,267]. The transparency of the films allows for constant observation of the wound, while their non-adherence to the skin surface prevents discomfort for the patient and ensures painless removal [269].

Transparent silicone membranes (Hyalomatrix^®^) containing a bilayered esterified HA may also be used for wound healing. They provide a protective and flexible cover for wounds and control water vapor loss. They are used for dressing pressure ulcers, second-degree burns, diabetic ulcers, and surgical and traumatic wounds [245,269]. In a study involving 10 participants with hard-to-heal wounds, the effectiveness of Hyalomatrix was evaluated. It was shown to reduce inflammatory symptoms in all patients [270].

#### 4.1.4. HA Creams and Gels for Wounds

HA is also a common ingredient in creams used for the treatment of wounds and burns [266,268]. HA creams provide a moist environment within the wound, support cell migration, and promote faster regeneration of damaged tissue [256]. It has been shown that the use of a 0.2% sodium salt of HA with 1% silver sulfadiazine (Connettivina Plus^®^) improves the healing of second-and third-degree pressure ulcers afflicting patients with chronic lesions [256,271]. In a randomized clinical trial, the effectiveness of Connettivina Plus was compared to that of Fitostimoline^®^ cream, which contained wheat extract as its main ingredient. Both preparations were shown to be effective in the treatment of acute superficial wounds, with better results observed for Fitostimoline. Both preparations also had high safety profiles [272].

HA gels used for wounds typically contain 2.5% sodium hyaluronate (Hylase^®^). They are suitable for exudative wounds prone to bleeding and minor cuts. They prevent tissue dehydration and support the wound-healing process [256].

## 5. Conclusions and Prospects

In this review, we have summarized the current knowledge regarding the role of hyaluronic acid (HA) in skin aging and wound healing and presented the latest applications of HA in cosmetology, aesthetic medicine, and regenerative medicine. HA is a common ingredient in many types of cosmetics, dietary supplements, tissue fillers, and wound dressings. In cosmetics, HA exhibits anti-wrinkle and moisturizing effects. However, HA’s impact on the skin depends on its molecular weight. HMW-HA does not penetrate the deeper layers of the skin but forms a protective film that prevents transepidermal water loss (TEWL) and has moisturizing properties. LMW-HA penetrates the dermis better, which is why it exhibits anti-wrinkle effects. HA is increasingly being used in dietary supplements aimed at improving skin quality. However, few studies have evaluated the effectiveness and safety of oral HA supplementation. Randomized clinical trials are needed to confirm the effects and safety of oral HA supplementation. Determining the optimal dose and molecular weight of HA for improving skin hydration and elasticity is also essential. HA is a key ingredient in fillers and skin biostimulators in aesthetic medicine. HA-based fillers have a high safety profile and are becoming more widely used for aesthetic purposes. HA biostimulators represent a promising new approach to skin rejuvenation, acting by stimulating fibroblasts to produce collagen. However, there is a limited number of studies evaluating the effectiveness of HA-based skin biostimulators in influencing neocollagenesis. Further studies, including controlled clinical trials, are necessary to confirm HA’s stimulating effect and evaluate the long-term effects of HA biostimulators on the skin. HA is also widely used in wound healing. It influences the migration of keratinocytes and fibroblasts, which are crucial in the healing process; enables the maintenance of a moist environment; and stimulates growth factors. Advances in technology have led to the development of innovative HA-based dressings, such as Hyaff-11 scaffolds and hydrogels, the effectiveness of which has been confirmed in clinical trials. HA has also become a popular material for targeted drug delivery systems.

The prospects for HA in regard to aging and wound-healing processes are promising. HA’s wide range of properties, particularly its biocompatibility and biodegradability, have made it a key polymer in regenerative medicine, cosmetics, and tissue engineering. Intensive research into making chemical modifications of HA to improve enzymatic degradation rates, biological integration, and biomechanical properties has expanded its applications in various biomedical fields. Innovations in HA applications, such as new delivery systems for drugs and active ingredients, are continually emerging. However, further research is needed to expand knowledge related to its cellular and molecular mechanisms and optimization for the application of HA in the skin. Advances in HA research could significantly improve patients’ aesthetic and health outcomes in the future.

## Figures and Tables

**Table 1:** Molecular weight-dependent biological activity of hyaluronic acid (HA).

Type of HA	Molecular Weight (kDa)	Biological Properties	References
High molecular weight	≥1000 kDa	Anti-inflammatory	[37,49,55]
		Anti-angiogenic	
		Immunosuppressive	
Medium molecular weight	250–1000 kDa	Proangiogenic	[51,58,59]
		Proinflammatory	
		Anti-apoptotic	
Low molecular weight	10–250 kDa	Proangiogenic	[5,51,60]
		Proinflammatory	
		Immunostimulatory	
HA oligomers	<10 kDa	Proangiogenic	[5,55,58]
		Proinflammatory	
		Highly immunostimulatory	
		Cell proliferation stimulatory	

**Table 2:** Examples of cosmetics with hyaluronic acid depending on the size of the molecule.

Types	kDa	Place of Action	Mechanism of Action	Skin Care Products			
				Creams	Serums	Masks	Tonics
High Molecular Weight	>1000 kDa	Stratum corneum	• Increases the viscosity of cosmetic products • Remains on the surface of skin • Serves a barrier function • Improves the hydration of the upper layers of the epidermis [134,143,148,154,155,156]	• Indeed Labs™ Hydraluron Moisture Jelly • Lierac Hydragenist The Rehydrating Radiance Cream • Sesderma Hidraderm Hyal Face Cream • By Terry Hyaluronic Global Face Cream • Skin&Lab Hybarrier Hyaluronic Cream • BERGAMO Hyaluronic Acid Essential Intensive Cream • L’Erbolario Triple Action Face Cream	• Unlëss Cosmetics Antioxidant Glow OxiShield Serum • Dermalure Hyaluronic Serum • L’Oreal Paris Revitalift Filler 1.5% Pure Hyaluronic Acid Serum • Mesoestetic^®^ HA Densimatrix Serum	• WIS+ Hyaluronic Acid Face Mask • L’Oréal Paris Hyaluron Specialist Tissue Mask • L’Erbolario Triple Action Face Mask • Germaine de Capuccini Timexpert Hydraluronic Hydra Nourishing Mask • The Organic Pharmacy Hyaluronic Acid Corrective Mask	• Purlés 160 Hydra Spray Toner • CLARENA^®^ Hyaluron 3D Tonic • Incarose Hyaluronic Tonic • Isntree^®^ Hyaluronic Acid Toner Plus • BERGAMO Hyaluronic Acid Intensive Toner
Medium Molecular Weight	250–1000 kDa	Epidermis	• Hydrates the epidermis • Improves wound healing [14,134,143,148]	• By Terry Hyaluronic Global Face Cream • Institut Esthederm Intensive Hyaluronic Cream • BERGAMO Hyaluronic Acid Essential Intensive Cream • L’Erbolario Triple Action Face Cream	• Mesoestetic^®^ HA Densimatrix Serum • Kokie Professional Hyaluronic Acid 2% Middle Molecular Weight Serum	• L’Erbolario Triple Action Face Mask • Germaine de Capuccini Timexpert Hydraluronic Hydra Nourishing Mask • Jalupro^®^ Masks • The Organic Pharmacy Hyaluronic Acid Corrective Mask	• Incarose Hyaluronic Tonic • Isntree^®^ Hyaluronic Acid Toner Plus • BERGAMO Hyaluronic Acid Intensive Toner
Low Molecular Weight	10–250 kDa	Dermis	• Improves skin hydration • Reduces wrinkles [5,134,143,148,157,158,159]	• By Terry Hyaluronic Global Face Cream • Sesderma Hidraderm Hyal Face Cream • Torriden DIVE-IN Low Molecule Hyaluronic Acid Cream • BERGAMO Hyaluronic Acid Essential Intensive Cream • L’Erbolario Triple Action Face Cream	• Nano Recipe Low Hyaluronic Molecular Acid 1% Serum • Cos De BAHA LMW HA Teca Serum • L’Oreal Paris Revitalift Filler 1.5% Pure Hyaluronic Acid Serum • Torriden DIVE-IN Hyaluronic Acid Serum • Mesoestetic^®^ HA Densimatrix Serum	• WIS+ Hyaluronic Acid Face Mask • L’Oréal Paris Hyaluron • Specialist Tissue Mask • Germaine de Capuccini Timexpert Hydraluronic Hydra Nourishing Mask • The Organic Pharmacy Hyaluronic Acid Corrective Mask • L’Erbolario Triple Action Face Mask	• CLARENA^®^ Hyaluron 3D Tonic • Torriden DIVE-IN Low Molecule Hyaluronic Acid Toner • Oliwia Plum Dash Tonic • Lantale Hyaluronic Acid Face Toner • Incarose Hyaluronic Tonic • Isntree^®^ Hyaluronic Acid Toner Plus
Ultra-Low Molecular Weight	<10 kDa	Dermis	• Penetrates the deeper layers of the skin • Improves skin hydration [148,160]	• Dulàc Anti Wrinkle Face Cream	• Isntree^®^ Ultra-low Molecular Hyaluronic Acid Serum • The Lab By Blanc Doux^®^ Oligo Hyaluronic Acid Hydro Serum	• Wellage Real Hyaluronic Blue Ampoule Face Mask	• Isntree^®^ Ultra-Low Molecular Hyaluronic Acid Toner

## Data Availability

Data sharing is not applicable to this article as no new data were created or analyzed in this study.
